# Testosterone therapy in hypogonadal men results in sustained and clinically meaningful weight loss

**DOI:** 10.1111/cob.12022

**Published:** 2013-06-19

**Authors:** AA Yassin, G Doros

**Affiliations:** 1 Institute of Urology and Andrology, Segeberger Kliniken, NorderstedtGermany; 2 Department of Epidemiology and Statistics, Boston University School of Public HealthBoston, MA, USA

**Keywords:** Testosterone, obesity, waist circumference, weight loss

## Abstract

**What is already known about this subject:**

- Hypogonadism is associated with increased fat mass and reduced muscle mass, which contributes to obesity and health risks, such as cardiovascular disease.
- Testosterone treatment of hypogonadal men improves muscle mass and reduces fat mass; however, many of these studies are of short duration.
- Thus, the long-term effects of testosterone on body anthropometry are not known.

**What this study adds:**

- Long-term testosterone treatment of hypogonadal men, up to 5 years duration, produced marked and significant decrease in body weight, waist circumference and body mass index.

Hypogonadism contributes to reduced muscle mass and increased adiposity. Testosterone treatment ameliorates loss of muscle mass and reduces fat accumulation associated with hypogonadism. In this study, we evaluated the long-term effects of normalizing testosterone (T) levels in hypogonadal men on anthropometric parameters. Open-label, single-center, cumulative, prospective registry study of 261 men (32–84 years, mean 59.5 ± 8.4 years, with T levels ≤12 nmol L^−1^ [mean: 7.7 ± 2.1]). Among the 261 men on T treatment, we followed up on 260 men for at least 2 years, 237 for 3 years, 195 for 4 years and 163 for at least 5 years. Subjects received parenteral T undecanoate 1000 mg every 12 weeks after an initial interval of 6 weeks. Body weight (BW), waist circumference (WC) and body mass index (BMI) were measured at baseline and yearly after treatment with T. BW decreased from 100.1 ± 14.0 kg to 92.5 ± 11.2 kg and WC was reduced from 107.7 ± 10.0 cm to 99.0 ± 9.1 cm. BMI declined from 31.7 ± 4.4 m kg^−2^ to 29.4 ± 3.4 m kg^−2^. All parameters examined were statistically significant vs. baseline and vs. the previous year over 5 years, indicating a continuous weight loss (WL) over the full observation period. The mean per cent WL was 3.2 ± 0.3% after 1 year, 5.6 ± 0.3%, after 2 years, 7.5 ± 0.3% after 3 years, 9.1 ± 0.3% after 4 years and 10.5 ± 0.4% after 5 years. The data obtained from this uncontrolled, observational, registry study suggest that raising serum T to normal physiological levels in hypogonadal men produces consistent loss in BW, WC and BMI. These marked improvements were progressive over the 5 years of the study.

## Introduction

Hypogonadism is characterized by low-serum T levels and a host of clinical symptoms including diminished libido and vitality, reduced muscle mass, erectile dysfunction, increased adiposity, depressed mood, osteopaenia, and osteoporosis 1–5. A substantial proportion of hypogonadal men may become overweight or obese and/ or diabetic 3,4. The increased adiposity and potential insulin resistance contribute to the risk factors of cardiovascular disease 5–6 and T treatment may ameliorate some of these risk factors 7–8. In previous studies, hypogonadal men treated with T, albeit for a short duration, showed increased muscle mass and reduced fat mass and reduced levels of serum low-density lipoprotein cholesterol (LDL-C) improved, blood pressure and heart rate (reviewed in 4–9). Abdominal obesity in hypogonadal men may be associated with reduced plasma T levels 10–11. In centrally obese individuals, there is an overactivity of the corticotropin-releasing hormone – adrenocorticotropin hormone – cortisol axis 12–13 and significant improvement in body composition was noted in several studies with T treatment 4,14, 15.

T plays a critical role in regulating energy utilization including nitrogen retention, carbohydrate and fat metabolism and adipogenesis. Androgens regulate differentiation of human mesenchymal stem cells and pre-adipocytes from different fat depots 16. Androgen deprivation induces the appearance of small multilocular adipocytes and T replacement restored normal adipocyte size and a unilocular phenotype in male white adipose tissue 17.

White adipose tissue from castrated animals exhibited evidence of altered morphological organization, impaired insulin response and reduction in expression of specific genes involved in regulation of energy use and adipogenesis. T replacement of castrated animals restored a normal unilocular white adipose tissue phenotype. Maneschi *et al*. 18 reported that T ameliorated the metabolic profile and reduced visceral adipose tissue in a high-fat diet-induced rabbit model of metabolic syndrome (MetS). Androgens regulate hormone-sensitive lipase activity in adipose tissue and inhibit adipogenesis. In hypogonadal men, T therapy leads to reduced abdominal fat 4, 19–24 and improvement in glucose-insulin homeostasis 25. Low-circulating androgen levels are thought to be associated with increased abdominal adiposity, and restoration of physiological levels leads to reduced abdominal fat.

Loss of fat mass and increase in lean mass was observed in hypogonadal men treated with T in a number of studies 15,22–26, but weight loss (WL) was only reported in two studies 27,28. One potential mechanism by which T contributes to WL is stimulating the basal metabolic rate, and increased physical activity, thus, resulting in WL. The role of T in modulating energy utilization such as increased nitrogen retention and increased carbohydrate and fat metabolism as well as regulation of adipogenesis has been recognized for some time 8,29–31.

T promotes rapid turnover of triglycerides by reducing triglyceride uptake and lipoprotein lipase activity, thus mobilizing lipids from the visceral fat depot and contributing to reduced fat mass. T may exert a direct and short-term effect on metabolism with an acute improvement in insulin sensitivity that occurs rapidly within a few days to a few weeks of treatment, and before loss of fat mass becomes evident; and a prolonged effect achieved when a significant reduction of total and visceral body fat occurs 26,32–34. Given that T replacement promotes a more active lifestyle, the rapid effects may be needed to prepare the body to greater energy expenditure 4. We believe that T treatment may bring about behavioural changes in addition to the increases in lean body mass, which contributes to increased energy expenditure. Over time, the increase in lean body mass would plateau and the decrease in fat mass will continue and this will bring about additional changes in weight. Thus, the findings of this study can be explained, in part, by the increase in lean body mass and reduction in fat mass attributed to changes in metabolism modulated by T treatment and the changes in physical activity. In addition, T treatment increases motivation, enhances mood and promotes more active lifestyle, thus preparing the body for physical activity and increased energy expenditure, thus contributing to further WL.

Although the treatment of hypogonadism with T remains somewhat controversial, management of hypogonadal men with T has received considerable attention and several T formulations are approved for the management of hypogonadal men. Treatment of hypogonadal men with T demonstrated considerable health benefits. However, the major criticism is that many of the studies on T therapy are of short duration. In this study, we investigated the effects of T treatment in a registry comprised of 261 hypogonadal men seeking urological consultation in a single urologist’s office for erectile dysfunction. Here, we report on the long-term effects of T treatment for up to 5 years on body weight (BW), waist circumference (WC) and body mass index (BMI) in hypogonadal men.

## Methods and procedures

We performed a cumulative registry study of 261 mainly elderly men, aged 32–84 years (mean ± standard deviation [SD] = 59.5 ± 8.4). All subjects had sought urological consultation in a single urologist’s office for erectile dysfunction. In addition to history and physical examinations, plasma T levels for each subject were measured by automated chemiluminescent microparticle immunoassay (Architect Abbott Diagnostics, Abbott Park, IL, USA); intra-assay coefficient of variability (CV) was 3.4% and inter-assay CV was 5.1%. Upon clinical and laboratory investigation, the subjects were found to have subnormal plasma total T levels (mean: 7.7 ± 2.1; range: 1.4–11.8 nmol L^−1^). All men received treatment with parenteral T undecanoate 1000 mg (Nebido®, Bayer Pharma, Berlin, Germany), administered at baseline and 6 weeks and thereafter every 12 weeks for up to 60 months.

Although there is no international consensus as to the normal range of testosterone, clinical data suggest that the normal range of T in adult men is between 12 and 40 nmol L^−1^ 35. A threshold of 12.1 nmol L^−1^ was recently confirmed by Bhasin *et al*., in an analysis of a number of important studies such as Framingham Heart Study generations 2 and 3, European Male Aging Study, and the Osteoporotic Fractures in Men Study 36.

Measurements of anthropometric parameters were performed at baseline (height, weight, WC) and at least once a year (weight, WC) and blood samples drawn at least once a year and prior to the next injection of testosterone. Therefore, T levels were trough levels at the end of an injection interval. WC was measured midway between the lowest rib and the uppermost border of the right iliac crest.

All 261 subjects in this registry were followed for at least one year, 260 subjects were followed for 2 years, 237 subjects were followed for 3 years, 195 subjects were followed for 4 years and 163 subjects were followed for 5 years. The number of subjects presented in the figures may seem to decrease over time. This is not due to the drop-out of subjects from the study, but rather because of the cumulative nature of the registry study design. New subjects were entered into the database once they had received 1 year of treatment with T. The declining number of patients over time therefore reflects duration of treatment but not drop-out rates. On the contrary, adherence to treatment was excellent, and T treatment was discontinued in only eight men, six of whom were diagnosed with prostate cancer and two men did not continue treatment for unknown reasons.

### Statistical analyses

Data included in the analyses are yearly data, which were obtained by averaging available visit data across each visit year. Each patient had approximately four visits per year. For continuous variables, the mean, median, SD, range, minimum, maximum, and sample size for the overall sample and various groups was reported at each time point. For categorical variables the frequency distribution was reported. We tested the hypotheses regarding change in outcome scores across the study period by fitting a linear mixed effects model to the data. Time (to indicate follow-up interviews) was included as fixed effect in the model. A random effect was included in the model for the intercept. Estimation and test of change in scores were determined by computing the differences in least square means at baseline vs. the score at each follow-up interview. For the correlation study, Pearson correlation was calculated between baseline changes in outcomes at various time points. The significance of each correlation was tested using Fisher’s test.

## Results

As shown in Table 1, the distribution of subjects based on BMI was: 11 men (4%) had normal weight (BMI ≤ 24.9), 88 men (34%) were overweight (BMI 25–25.9), 162 men (62%) were obese (BMI ≥ 30) at baseline. In this latter subgroup, 152 men (94%) were obese (BMI 30–39.9) and 10 men (6%) were excessively obese (BMI ≥ 40). The distribution based on WC was: 8 men (3%) had normal WC of <94 cm, 74 men (28%) had WC of 94–101.9 cm and 179 men (69%) men had WC greater than 102 cm. All men had presented with erectile function problems, and the majority of men had a host of known comorbidities at baseline (Table 1).

**Table:** Baseline Characteristics of Patients in the Registry

Mean age (years)	59.5 ± 8.4	
Parameter	*n*	Proportion (%)
BMI category		
Normal weight (BMI ≤24.9)	11	4
Overweight (BMI 25–25.9)	88	34
Obese (BMI 30+)	162	62
Waist circumference category		
Normal (<94 cm)	8	3
Increased (94–101.9 cm)	74	28
Substantially increased (≥102 cm)	179	69
Known comorbidities at baseline		
Hypertension	117	45
Type 2 diabetes	80	31
Dyslipidaemia	87	33
Coronary artery disease	32	12
Erectile dysfunction	261	100
BPH/LUTS	150	57
Prostatitis	30	11
Osteoporosis	14	5

BMI, body mass index; BPH, benign prostatic hyperplasia; LUTS, lower urinary tract symptoms.

### Total T levels during the 60 months period of treatment

**Figure 1:**
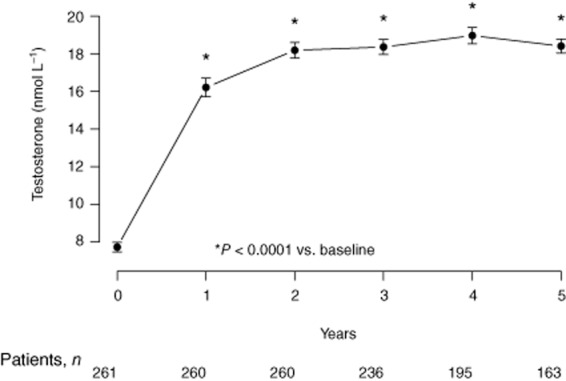
Total serum testosterone levels in hypogonadal men over the course of 60 months of testosterone treatment.

Treatment of hypogonadal men with T for up to 60 months resulted in a significant increase in total T levels (Fig. 1). Total T levels rose from 7.72 ± 2.07 nmol L^−1^ at beginning of T therapy to 16.2 ± 4.1 nmol L^−1^ within the first 12 months of T therapy and these values stabilized between 18 and 19 nmol L^−1^ for the remainder of the observation period.

### Treatment of hypogonadal men with T produced reduction in WC

**Figure 2:**
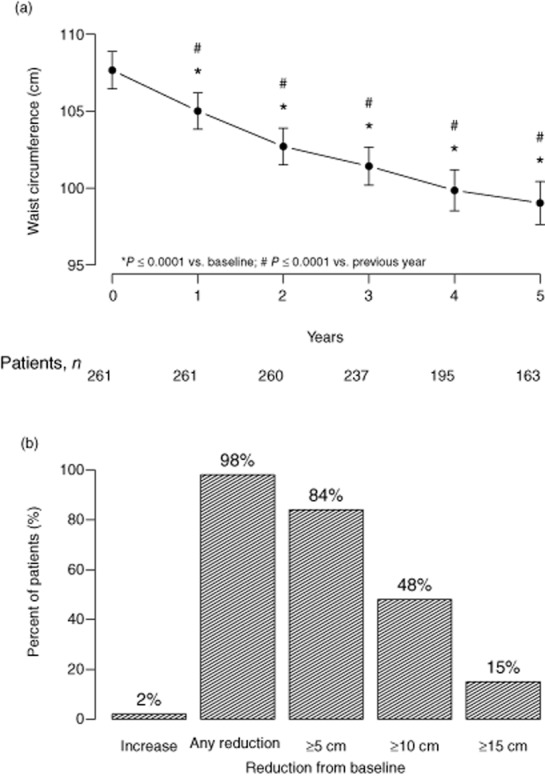
Reduction in waist circumference (cm) in hypogonadal men in response to testosterone treatment (a); per cent of patients with varying degrees of waist circumference reduction (b).

In hypogonadal men treated with T, WC declined from 107.7 ± 10.0 cm (min 88, max 148) to 99.0 ± 9.0 cm (min 85, max 137) with a mean reduction of 9.4 ± 0.3 cm (*P* < 0.0001; Fig. 2a). The reduction in WC was statistically significant at the end of each year compared to the previous year (*P* < 0.0001) over the full 5-year observation period. In this study, 97.5% of all men showed a decrease in WC. WC decreased by ≥5 cm in 84% of men and by ≥10 cm in 48% of men. Approximately 15% of men had a decrease in WC by ≥15 cm and only 2% had an increase in WC (Fig. 2b).

### Treatment of hypogonadal men with T produced significant weight loss

**Figure 3:**
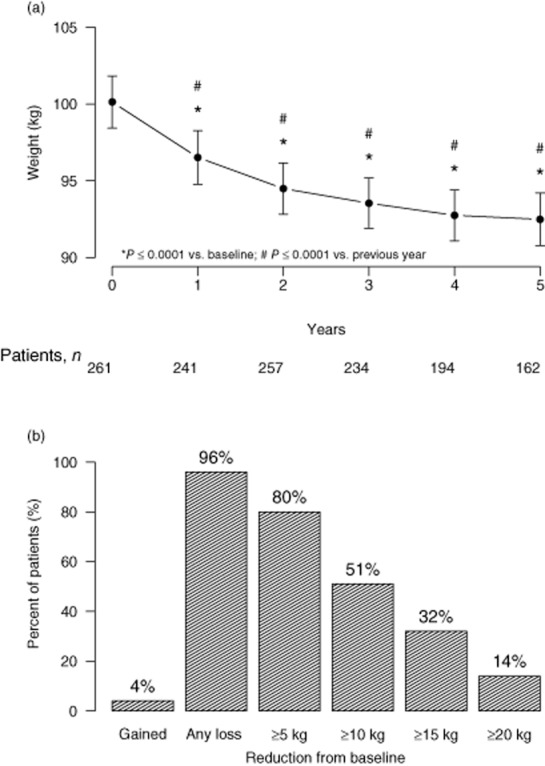
Reduction in weight (kg) in hypogonadal men in response to testosterone treatment (a); per cent of patients with varying degrees of weight loss (b).

Figure 3A shows the effects of T therapy on BW in hypogonadal men over the course of 5 years of treatment. Weight decreased from 100.1 ± 14.0 kg (minimum: 68, maximum: 141) to 92.5 ± 11.2 kg (min 67, max 124) with a mean loss of 11.1 ± 0.4 kg. This decrease in BW was statistically significant at the end of each year compared with the previous year (*P* < 0.0001) over the full 5-year observation period. We noted that 14% of men lost ≥20 kg, 31% of men lost ≥15 kg, 51% of men lost ≥10 kg and 80% of men lost ≥5 kg over the 5 years period of T therapy. Approximately 4% of men gained some weight (Fig. 3B).

### Percentage change in BW as a result of treatment of hypogonadal men with T

**Figure 4:**
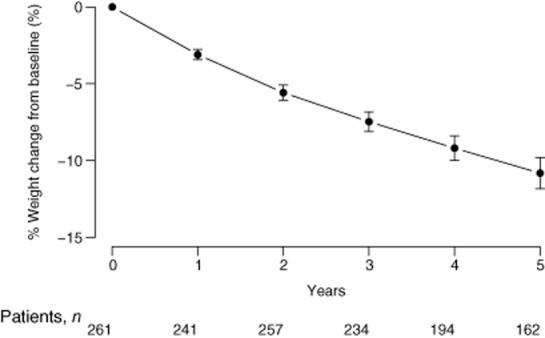
Per cent weight loss from baseline in hypogonadal men receiving testosterone treatment over the course of 60 months.

Marked and significant decrease in percentage BW was noted over the course of T treatment. Over the entire observation period, patients lost 10.5% of their initial BW (Fig. 4). Patients had lost 3.2 ± 0.3% of their initial weight after 1 year, 5.6 ± 0.3% after 2 years, 7.5 ± 0.3% after 3 years, 9.1 ± 0.3% after 4 years and 10.5 ± 0.4% after 5 years. These changes were statistically significant at the end of each year compared with the previous year (*P* < 0.0001) over the full 5-year observation period. When correlating per cent weight change with baseline T, we found weak correlation across the 5 years of follow-up.

### Treatment of hypogonadal men with T produced significant decline in BMI

**Figure 5:**
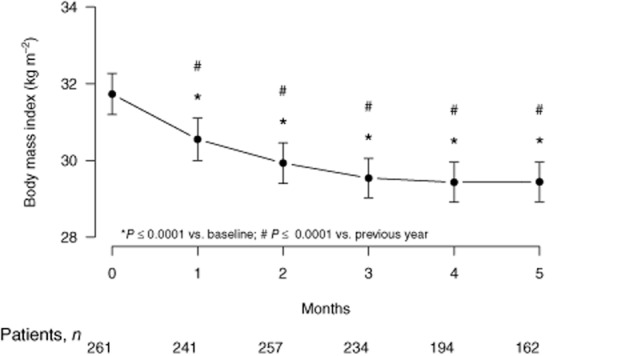
Body mass index (BMI, kg m^−2^) in hypogonadal men in response to testosterone treatment over the course of 60 months.

Over the entire course of 60 months of treatment, we noted consistent and progressive decline in BMI (Fig. 5). BMI declined from 31.7 ± 4.4 to 30.6 ± 4.3 after 1 year, 29.9 ± 4.3 after 2 years, 29.5 ± 4.0 after 3 years, 29.4 ± 3.7 after 4 years and 29.4 ± 3.4 after 5 years. The observed decline in BMI was consistent with the recorded reductions in WC and weight.

**Figure 6:**
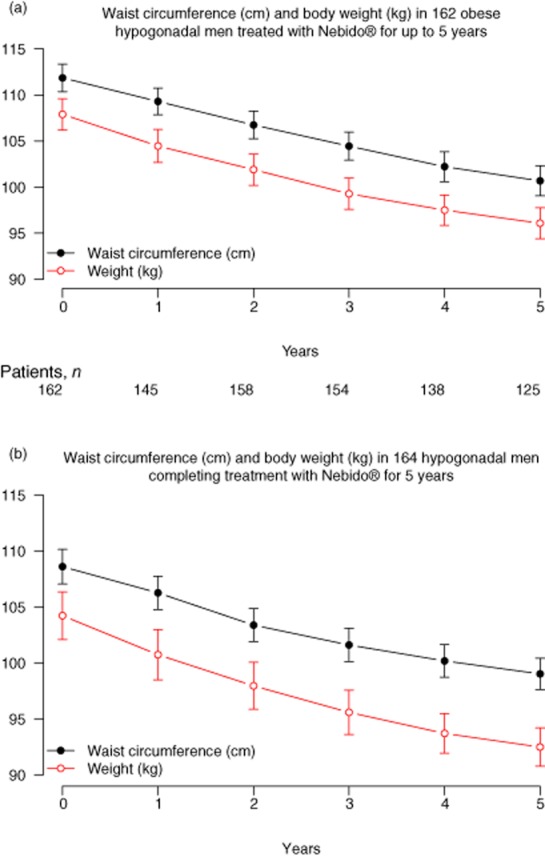
Subgroup analysis of waist circumference (cm) and body weight (kg) in 162 obese hypogonadal men treated with T undecanoate for 5 years (a); analysis of waist circumference (cm) and bodyweight (kg) in 164 hypogonadal men who completed 5 years of T undecanoate treatment (b).

The data were further analysed to determine the changes in weight, WC and BMI in men who were obese at baseline. One hundred sixty-two men (62%), in this cohort, had had a BMI of 30 or higher at baseline. In this subgroup with a mean age of 59.8 ± 7.9 years, BW declined from 107.9 ± 11.1 to 96.1 ± 9.5 kg after 5 years. The mean WL in this subgroup was 12.8 ± 0.5 kg. WC decreased from 111.9 ± 9.8 to 100.7 ± 9.3 cm with a mean reduction of 10.5 ± 0.3 cm, BMI was reduced from 34.5 ± 3.2 to 30.7 ± 2.7 kg m^−2^ (Fig. 6A). All three parameters remained statistically significant over the full observation time. Approximately 95% of the obese men lost some weight; 18% lost more than 20 kg, 40% lost more than 15 kg, 64% lost more than 10 kg, 84% lost more than 5 kg, and only 5% gained some weight. WC was reduced in 98% of the obese subgroup, 20% lost more than 15 cm, 55% more than 10 cm, 89% more than 5 cm and only 2% had an increase in WC.

In order to avoid a bias that may have occurred as a result of the registry design of our study, we also performed a subgroup analysis of those patients who had completed 5 years of treatment. One hundred sixty-four men fulfilled this criterion. In these ‘completers’ with a mean age of 59.6 ± 8.0 years, BW decreased from 104.2 ± 13.9 to 92.5 ± 11.2 kg by a mean of 11.7 ± 0.4 kg, WC diminished from 108.6 ± 10.1 to 99.0 ± 9.1 cm with a mean of 9.6 ± 0.3 cm (Fig. 6B), and BMI declined from 33.2 ± 4.3 to 29.4 ± 3.4 kg m^−2^. Approximately 96% of completers lost some weight, 14% lost ≥20 kg, 31% ≥15, 51% ≥10, 80% ≥5 kg and only 4% showed some weight gain.

**Figure 7:**
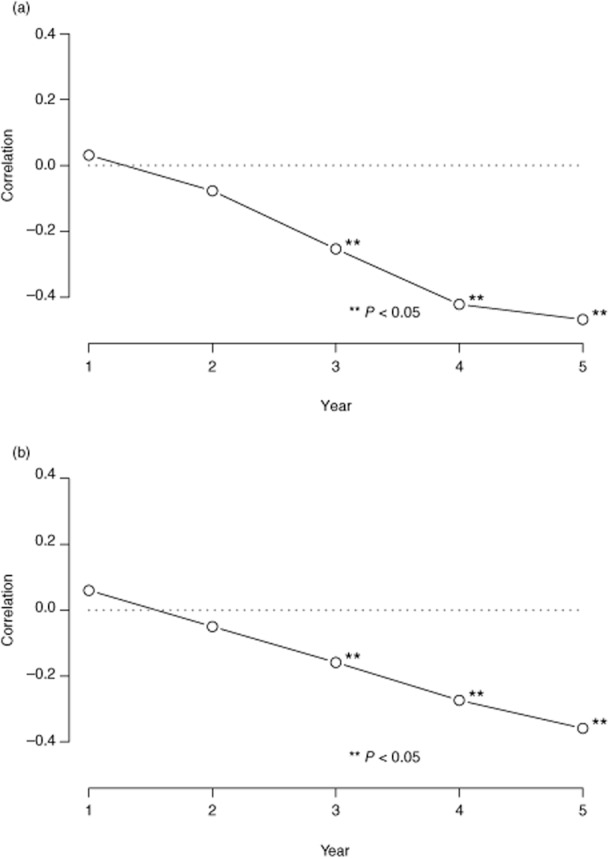
Pearson’s correlation between weight at baseline and changes in waist circumference, over the 5 years period of T treatment (a); Pearson’s correlation between weight at baseline and changes in weight over the course of 5 years of treatment (b).

In an attempt to determine the relationship between weight at baseline and changes in weight and WC, over the 5-year period of T treatment, we analysed the data based on Pearson’s correlation. As shown in Fig. 7, there were significant correlations between baseline BMI and changes in weight and WC. These findings suggest that the loss of weight in response to T treatment is correlated with baseline BMI and this correlation becomes significant over the course of T treatment.

## Discussion

The data from this single site, uncontrolled, prospective registry study of 261 men seeking treatment for erectile dysfunction showed that T treatment of hypogonadal men produced marked WL in approximately 96% of all patients. T treatment produced significant, gradual and sustainable WL and this was associated with marked reductions in WC and BMI, suggesting that in hypogonadal men, T therapy brought about changes in body anthropometry consistent with previous findings demonstrating increase in lean body mass and decrease in fat mass in men treated with T (reviewed in 4). T treatment also produced consistent and marked reductions in WC and BMI over the course of 60 months of treatment and follow-up. Subgroup analysis indicated that the magnitude of changes in the aforementioned parameters was more pronounced among the obese. Based on subgroup analysis, we noted a correlation between baseline weight and changes in weight and WC. Also we noted marked reductions in WC, weight and BMI in the overweight and obesity classes I, II and III, suggesting that T treatment results in changes in anthropometric parameters in all these subgroups (data not shown). The findings of this study were unexpected and surprising, as it was not designed to investigate the effects of T on weight in hypogonadal men.

Obesity reduces T levels in men, via various mechanisms 4. The prevalence of T deficiency in obese men ranges from 29.3% to 52.6% 37,38. Decreased fat mass and increased lean body mass is the most common reported observation in hypogonadal men treated with T. The minimal to moderate WL reported in previous studies are attributed in part to the short durations 21–24 and only few studies using long-acting injections of T undecanoate had durations of two or more years 15,26,27. In a pilot, placebo-controlled study in men with MetS treated for 3 years with T undecanoate, WC declined by 13 cm 15. These findings suggest that T is an important physiological modulator of body composition because of its role in functional fuel metabolism and in promoting myogenesis and inhibiting adipogenesis.

Longitudinal population studies suggested that low T is an independent risk factor for the development of MetS and type 2 diabetes, stroke or transient ischemic attacks. An inverse relationship between T levels and indicators of obesity (BMI, WC), has been reported 5. T treatment of men with the MetS produced moderate weight reduction and reduced insulin resistance 3.

The marked WL reported in this study is attributed to changes in metabolism in response to normalization of T levels. T regulates metabolic function and energy utilization resulting in nitrogen retention, carbohydrate and fat metabolism, and regulation of adipogenesis 8,9. T promotes rapid turnover of triglycerides in the subcutaneous abdominal adipose tissue and mobilizes lipids from the visceral fat depot by inhibiting triglyceride uptake and lipoprotein lipase activity. Furthermore, T regulates differentiation of mesenchymal pluripotent cells and promotes the myogenic lineage and inhibits the adipogenic lineage 39. The reported increase in lean body mass and reduction in fat mass, in previous studies, suggest that changes in metabolism are modulated by T treatment. Thus, our findings may be explained in part by changes in metabolism contributing to WL and reduction in WC and reductions in body mass index. Our findings are similar to that of a recently published report in which T treatment produced significant reduction in weight and marked decrease in BMI and WC 27.

A recent study 40 demonstrated that, in ageing men, moderate WL was associated with an increase in testosterone and more substantial WL was associated with an increase in free T. These findings suggested that health and lifestyle factors contribute to WL with profound effects on the function of hypothalamic pituitary testicular axis in ageing men. Such factors attenuate or reverse the apparent age-related testosterone-decline. The implication of such findings is that maintaining the function of the hypothalamic pituitary testicular axis, in ageing, may require reducing weight gain and treating obesity. These findings support the data presented in this study in that T plays an important and direct role in regulating the anthropometric parameters in aging men.

In a recent International Post-Authorisation Surveillance Study (IPASS) of more than 1400 hypogonadal men from 155 centres in 23 countries the overall level of vigour/vitality was markedly increased after 1 year of T treatment 41. This, together with improvement in the quality of life, explains, in part, the possible increase in physical activity subsequent to T treatment. Furthermore, the increased motivation, level of energy and vigour associated with T treatment may explain the marked reduction in BW attributed to increased energy expenditure.

As reported previously, it should be noted that one of the major concerns regarding T treatment in hypogonadal men is prostate cancer 27. To date, there is no compelling evidence that T promotes development of prostate cancer in men 42,43 and guidelines for monitoring have been developed which, if properly utilized, renders T treatment as safe in management of hypogonadal men without fear of prostate carcinoma 1,45–48. In this study of 261 patients treated with T for 5 years, only six patients had a diagnosis of prostate cancer, representing an incidence per 10 000 patients years of 54.4, a smaller incidence than reported in the Prostate, Lung, Colorectal, Ovarian Cancer Screening Trial 49 and in the European Randomized Study of Screening for Prostate Cancer Patients 50. The data from this study can only suggest that there was no signal of an increased risk of prostate cancer.

Among the limitations of this study is the nature of its registry design. This single-centre, open-label study is not a randomized controlled study and therefore limits interpretation of the reported findings. Subjects were treated in a urology clinical setting for a host of urological conditions including benign erectile dysfunction, prostatic hyperplasia and lower urinary tract symptoms. Because these subjects are seeking medical treatment of various urological conditions, an unintended bias may have been introduced. Another potential limitation of this study is that we only measured total T levels, but not free or bioavailable testosterone, in combinations with signs and symptoms, to assess hypogonadism. Further, patients in this registry exhibited different urological complaints and have different comorbidities. Because we did not expect patients to lose weight, there was no assessment of lifestyle changes. We cannot exclude that men adopted a more active lifestyle and increased their level of physical activity. It has been shown that treatment of hypogonadal men with long-acting injections of T undecanoate increases vigour and vitality 41,51.

It should be noted that this study was not designed to investigate WL, and patients were entered into the registry consecutively once diagnosed with hypogonadism and had received at least one year of T treatment. This may have introduced a bias of some patients dropping out in the early treatment phase because of lack of response. Another bias may have been introduced by the fact that patients treated for less than 5 years may have had different results. A subgroup analysis of those men who had completed 5 years of treatment, however, revealed that the changes were in the same magnitude as in the total group.

In this study, T was used to treat hypogonadal men and the effects of T treatment on weight was neither intended nor expected, as long-term WL had never before been reported in the literature as an outcome measure for T treatment. In one study, men with MetS lost 4.31 kg BW after 30 weeks of treatment with injectable T undecanoate 27. In this study, there was a marked and significant WL of approximately 11 kg in 96% of subjects). In summary, T treatment in hypogonadal men for up to 5 years duration appears to be effective in facilitating WL, reduction in WC and BMI.

## Conflict of interest statement

No conflict of interest was declared.

## Disclosure

Aksam Yassin has received honoraria for lectures from Bayer Healthcare, Germany, Ferring Pharmaceuticals, Germany, and GSK, Germany. Gheorghe Doros has received compensation for statistical analyses from Bayer Healthcare, Germany.

## References

[1] Buvat J, Maggi M, Guay A, Torres LO (2013). Testosterone deficiency in men: systematic review and standard operating procedures for diagnosis and treatment. J Sex Med.

[2] Traish AM, Miner MM, Morgentaler A, Zitzmann M (2011). Testosterone deficiency. Am J Med.

[3] Wang C, Jackson G, Jones TH (2011). Low testosterone associated with obesity and the metabolic syndrome contributes to sexual dysfunction and cardiovascular disease risk in men with type 2 diabetes. Diabetes Care.

[4] Saad F, Aversa A, Isidori AM, Gooren LJ (2012). Testosterone as potential effective therapy in treatment of obesity in men with testosterone deficiency: a review. Curr Diabetes Rev.

[5] Traish AM, Saad F, Feeley RJ, Guay A (2009). The dark side of testosterone deficiency: III. Cardiovascular disease. J Androl.

[6] Corona G, Rastrelli G, Monami M (2011). Hypogonadism as a risk factor for cardiovascular mortality in men: a meta-analytic study. Eur J Endocrinol.

[7] Saad F (2012). Androgen therapy in men with testosterone deficiency: can testosterone reduce the risk of cardiovascular disease?. Diabetes Metab Res Rev.

[8] Traish AM, Kypreos KE (2011). Testosterone and cardiovascular disease: an old idea with modern clinical implications. Atherosclerosis.

[9] Traish AM, Guay A, Feeley R, Saad F (2009). The dark side of testosterone deficiency: I. Metabolic syndrome and erectile dysfunction. J Androl.

[10] Pasquali R, Casimirri F, Cantobelli S (1991). Effect of obesity and body fat distribution on sex hormones and insulin in men. Metabolism.

[11] Khaw KT, Barrett-Connor E (1992). Lower endogenous androgens predict central adiposity in men. Ann Epidemiol.

[12] Gapstur SM, Gann PH, Kopp P (2002). Serum androgen concentrations in young men: a longitudinal analysis of associations with age, obesity, and race. The CARDIA male hormone study. Cancer Epidemiol Biomarkers Prev.

[13] Björntorp P, Rosmond R (2000). The metabolic syndrome – a neuroendocrine disorder?. Br J Nutr.

[14] Behre HM, Tammela TL, Arver S (2012). A randomized, double-blind, placebo-controlled trial of testosterone gel on body composition and health-related quality-of-life in men with hypogonadal to low-normal levels of serum testosterone and symptoms of androgen deficiency over 6 months with 12 months open-label follow-up. Aging Male.

[15] Aversa A, Bruzziches R, Francomano D (2012). Effects of long-acting testosterone undecanoate on bone mineral density in middle-aged men with late-onset hypogonadism and metabolic syndrome: results from a 36 months controlled study. Aging Male.

[16] Gupta V, Bhasin S, Guo W (2008). Effects of dihydrotestosterone on differentiation and proliferation of human mesenchymal stem cells and preadipocytes. Mol Cell Endocrinol.

[17] Varlamov O, White AE, Carroll JM (2012). Androgen effects on adipose tissue architecture and function in nonhuman primates. Endocrinology.

[18] Maneschi E, Morelli A, Filippi S (2012). Testosterone treatment improves metabolic syndrome-induced adipose tissue derangements. J Endocrinol.

[19] Blouin K, Nadeau M, Perreault M (2010). Effects of androgens on adipocyte differentiation and adipose tissue explant metabolism in men and women. Clin Endocrinol (Oxf).

[20] Hamilton EJ, Gianatti E, Strauss BJ (2011). Increase in visceral and subcutaneous abdominal fat in men with prostate cancer treated with androgen deprivation therapy. Clin Endocrinol.

[21] Bhasin S, Travison TG, Storer TW (2012). Effect of testosterone supplementation with and without a dual 5α-reductase inhibitor on fat-free mass in men with suppressed testosterone production: a randomized controlled trial. JAMA.

[22] Srinivas-Shankar U, Roberts SA, Connolly MJ (2010). Effects of testosterone on muscle strength, physical function, body composition, and quality of life in intermediate-frail and frail elderly men: a randomized, double-blind, placebo-controlled study. J Clin Endocrinol Metab.

[23] Svartberg J, Agledahl I, Figenschau Y (2008). Testosterone treatment in elderly men with subnormal testosterone levels improves body composition and BMD in the hip. Int J Impot Res.

[24] Allan CA, Strauss BJ, Burger HG, Forbes EA, McLachlan RI (2008). Testosterone therapy prevents gain in visceral adipose tissue and loss of skeletal muscle in non-obese aging men. J Clin Endocrinol Metab.

[25] Mårin P, Holmäng S, Jönsson L (1992). The effects of testosterone treatment on body composition and metabolism in middle-aged obese men. Int J Obes Relat Metab Disord.

[26] Aversa A, Bruzziches R, Francomano D (2010). Effects of testosterone undecanoate on cardiovascular risk factors and atherosclerosis in middle-aged men with late-onset hypogonadism and metabolic syndrome: results from a 24-month, randomized, double-blind, placebo-controlled study. J Sex Med.

[27] Saad F, Haider A, Doros G, Traish A (2013). Long-term treatment of hypogonadal men with testosterone produces substantial and sustained weight loss. Obesity.

[28] Kalinchenko SY, Tishova YA, Mskhalaya GJ (2010). Effects of testosterone supplementation on markers of the metabolic syndrome and inflammation in hypogonadal men with the metabolic syndrome: the double-blinded placebo-controlled Moscow study. Clin Endocrinol.

[29] Traish AM, Abdou R, Kypreos KE (2009). Androgen deficiency and atherosclerosis: the lipid link. Vascul Pharmacol.

[30] Aub JC (1940). The use of testosterone. N Engl J Med.

[31] Aub JC, Kety SS (1943). Recent advances in testosterone therapy. N Engl J Med.

[32] Yialamas MA, Dwyer AA, Hanley E (2007). Acute sex steroid withdrawal reduces insulin sensitivity in healthy men with idiopathic hypogonadotropic hypogonadism. J Clin Endocrinol Metab.

[33] Pitteloud N, Mootha VK, Dwyer AA (2005). Relationship between testosterone levels, insulin sensitivity, and mitochondrial function in men. Diabetes Care.

[34] Aversa A, Bruzziches R, Francomano D, Spera G, Lenzi A (2010). Efficacy and safety of two different testosterone undecanoate formulations in hypogonadal men with metabolic syndrome. J Endocrinol Invest.

[35] Nieschlag E, Behre HM (1997). Andrology.

[36] Bhasin S, Pencina M, Jasuja GK (2011). Reference ranges for testosterone in men generated using liquid chromatography tandem mass spectrometry in a community-based sample of healthy nonobese young men in the Framingham Heart Study and applied to three geographically distinct cohorts. J Clin Endocrinol Metab.

[37] Mulligan T, Frick MF, Zuraw QC, Stemhagen A, McWhirter C (2006). Prevalence of hypogonadism in males aged at least 45 years: the HIM study. Int J Clin Pract.

[38] Corona G, Rastrelli G, Monami M (2011). Body mass index regulates hypogonadism-associated cv risk: results from a cohort of subjects with erectile dysfunction. J Sex Med.

[39] Singh R, Artaza JN, Taylor WE, Gonzalez-Cadavid NF, Bhasin S (2003). Androgens stimulate myogenic differentiation and inhibit adipogenesis in C3H 10T1/2 pluripotent cells through an androgen receptor-mediated pathway. Endocrinology.

[40] Camacho EM, Huhtaniemi IT, O’Neill TW (2013). Age-associated changes in hypothalamic-pituitary-testicular function in middle-aged and older men are modified by weight change and lifestyle factors: longitudinal results from the European Male Ageing Study. Eur J Endocrinol.

[41] Zitzmann M, Mattern A, Hanisch J (2013). IPASS: a study on the tolerability and effectiveness of injectable testosterone undecanoate for the treatment of male hypogonadism in a worldwide sample of 1,438 men. J Sex Med.

[42] Morgentaler A, Traish AM (2009). Shifting the paradigm of testosterone and prostate cancer: the saturation model and the limits of androgen-dependent growth. Eur Urol.

[43] Morgentaler A (2012). Goodbye androgen hypothesis, hello saturation model. Eur Urol.

[44] Muller RL, Gerber L, Moreira DM (2012). Serum testosterone and dihydrotestosterone and prostate cancer risk in the placebo arm of the reduction by dutasteride of prostate cancer events trial. Eur Urol.

[45] Bhasin S, Cunningham GR, Hayes FJ (2010). Testosterone therapy in men with androgen deficiency syndromes: an Endocrine Society clinical practice guideline. J Clin Endocrinol Metab.

[46] Wang C, Nieschlag E, Swerdloff RS (2009). ISA, ISSAM, EAU, EAA and ASA recommendations: investigation, treatment and monitoring of late-onset hypogonadism in males. Aging Male.

[47] Dohle GR, Arver S, Bettocchi C (2012). Guidelines on male hypogonadism. Eur Ass Urol.

[48] Morales A, Bain J, Ruijs A, Chapdelaine A, Tremblay RR (1996). Clinical practice guidelines for screening and monitoring male patients receiving testosterone supplementation therapy. Int J Impot Res.

[49] Andriole GL, Crawford ED, Grubb RL, PLCO Project Team (2009). Mortality results from a randomized prostate-cancer screening trial. N Engl J Med.

[50] Schröder FH, Hugosson J, Roobol MJ, ERSPC Investigators (2012). Prostate-cancer mortality at 11 years of follow-up. N Engl J Med.

[51] Tong S-F NC-J, Lee B-C (2012). Effect of long-acting testosterone undecanoate treatment on quality of life in men with testosterone deficiency syndrome: a double blind randomized controlled trial. Asian J Androl.

